# Genetic polymorphisms and risk of recurrent wheezing in pediatric age

**DOI:** 10.1186/1471-2466-14-162

**Published:** 2014-10-18

**Authors:** Susanna Esposito, Valentina Ierardi, Cristina Daleno, Alessia Scala, Leonardo Terranova, Claudia Tagliabue, Walter Peves Rios, Claudio Pelucchi, Nicola Principi

**Affiliations:** Pediatric Highly Intensive Care Unit, Department of Pathophysiology and Transplantation, Università degli Studi di Milano, Fondazione IRCCS Ca’ Granda Ospedale Maggiore Policlinico, Via Commenda 9, 20122 Milan, Italy; Department of Epidemiology, IRCCS Istituto di Ricerche Farmacologiche Mario Negri, Milan, Italy

**Keywords:** Asthma, Genetic polymorphisms, Lower respiratory tract infection, Recurrent wheezing, Respiratory viruses, Wheezing

## Abstract

**Background:**

Wheezing during early life is a very common disorder, but the reasons underlying the different wheezing phenotypes are still unclear. The aims of this study were to analyse the potential correlations between the risk of developing recurrent wheezing and the presence of specific polymorphisms of some genes regulating immune system function, and to study the relative importance of the associations of different viruses and genetic polymorphisms in causing recurrent episodes.

**Methods:**

The study involved 119 otherwise healthy infants admitted to hospital for a first episode of wheezing (74 of whom subsequently experienced recurrent episodes) and 119 age- and sex-matched subjects without any history of respiratory problem randomly selected from those attending our outpatient clinic during the study period. All of the study subjects were followed up for two years, and 47 single nucleotide polymorphisms (SNPs) in 33 candidate genes were genotyped on whole blood using an ABI PRISM 7900 HT Fast Real-time instrument.

**Results:**

IL8-rs4073AT, VEGFA-rs833058CT, MBL2-rs1800450CT and IKBKB-rs3747811AT were associated with a significantly increased risk of developing wheezing (p = 0.02, p = 0.03, p = 0.05 and p = 0.0018), whereas CTLA4-rs3087243AG and NFKBIB-rs3136641TT were associated with a significantly reduced risk (p = 0.05 and p = 0.04). IL8-rs4073AT, VEGFA-rs2146323AA and NFKBIA-rs2233419AG were associated with a significantly increased risk of developing recurrent wheezing (p = 0.04, p = 0.04 and p = 0.03), whereas TLR3-rs3775291TC was associated with a significantly reduced risk (p = 0.03). Interestingly, the study of gene-environment interactions showed that rhinovirus was significantly associated with recurrent wheezing in the presence of IL4Ra-rs1801275GG and G (odds ratio [OR] 6.03, 95% confidence interval [CI]: 1.21-30.10, p = 0.03) and MAP3K1-rs702689AA (OR 4.09, 95% CI: 1.14-14.61, p = 0.03).

**Conclusions:**

This study shows a clear relationship between the risk of wheezing and polymorphisms of some genes involved in the immune response. Although further studies are needed to confirm the results, these findings may be useful for the early identification of children at the highest risk of developing recurrent episodes and possibly subsequent asthma.

## Background

Wheezing during early life is a very common disorder: longitudinal birth cohort studies have estimated that its cumulative prevalence during pre-school years is approximately 50% [1]. In most cases, wheezing in infants and young children is associated with a viral acute lower respiratory tract infection (LRTI) mainly due to respiratory syncytial virus (RSV) or rhinovirus (RV), and less frequently to other viruses such as human metapneumovirus, bocavirus and parainfluenza virus [2].

Some children experience only one episode of wheezing or a very small number of infrequent recurrences, and are usually not affected by significant respiratory problems in later life. However, a substantial number of children experience wheezing every time they suffer from a respiratory viral infection, and up to 50% of these subjects develop asthma during school age [3]. The reasons for these different wheezing phenotypes are still unclear. A number of clinical and epidemiological studies have shown that the severity of individual episodes, the risk of recurrence, and the risk of developing asthma are frequently associated with the presence of atopy or particular clinical or environmental conditions such as prematurity and exposure to tobacco smoke [4, 5]. Microbiological studies have shown that not all viruses have the same potential for causing severe or recurrent cases, and that RSV [6] and RV [7] are more frequently identified in the respiratory secretions of subjects with recurrent wheezing. However, analysis of the data collected in studies of the possible relationships between viral LRTIs and wheezing has shown that, even in the absence of known risk factors, a considerable number of children experience severe and/or frequently recurring infections with wheezing, and develop asthma during adolescence [2].

It has been suggested that alterations in the innate and adaptive immune responses to viral LRTIs may favour virus-induced airway dysfunctions that lead to more severe signs and symptoms, and long-lasting changes in the anatomic and histological characteristics of the respiratory tree that predispose to recurrences and the development of asthma [8], and a number of studies indicate that these risks seem to be closely associated with genetic polymorphisms of the genes encoding factors involved in innate and adaptive immunity [9–11]. However, little is known about which immune response mechanisms modified by genetic defects play the major role in conditioning susceptibility to wheezing, or what can be done to increase host defences in such cases. The precise identification of the specific genetic variants involved in immunity that are associated with an increased risk of LRTIs with recurrent wheezing has foreseeable applications in pediatrics, preventive medicine and vaccine development, and it can be expected that analysing methods that can reduce the negative impact of genetic defects in innate or adaptive immunity will have considerable consequences.

The aims of this study were to analyse the potential correlations between the risk of developing recurrent LRTIs with wheezing and the presence of specific polymorphisms of some genes that regulates immune system function, and to study the relative importance of the associations of different viruses and genetic polymorphisms in causing recurrent episodes.

## Methods

### Study population and recruitment

The study was carried out in the Pediatric Highly Intensive Care Unit of the Fondazione IRCCS Ca’ Granda, Ospedale Maggiore Policlinico, Milan, Italy, between November 2010 and September 2013. The protocol was approved by the Ethics Committee of the Fondazione IRCCS Ca’ Granda, Ospedale Maggiore Policlinico, and written informed consent was obtained from the parents or legal guardian of each patient before enrolment.

The study initially involved 138 otherwise healthy infants aged ≤12 months admitted to hospital because of a first episode of RTI with wheezing diagnosed on the basis of well-established criteria [12]. In particular, were infants with clinical evidence of respiratory tract infection with fever, stuffed or runny nose, sneezing and/or cough accompanied by wheezing (i.e. a continuous high-pitched sound with musical quality emitting from the chest during expiration) were enrolled. The exclusion criteria were premature birth or the presence of a chronic disease increasing the risk of complications of a respiratory infection (i.e. a chronic pulmonary or cardiovascular system disorder, chronic metabolic disease, neoplasia, kidney or liver dysfunction, hemoglobinopathy, immunosuppression, and or genetic or neurological disorders). After selection, there was no refusal to participate. Upon enrolment, the demographic characteristics and medical history of the infants were systematically recorded using standardised written questionnaires, and specimens for the detection of respiratory viruses were taken using flexible pernasal flocked swabs and 1 mL of universal trasport medium (Kit Cat. No. 360c, Copan Italia, Brescia, Italy). At enrolment, a 3 mL whole blood sample was also obtained for genetic studies.

After enrolment, all of the children were followed up for 24 months in order to monitor the incidence of new wheezing episodes. To this end, the parents were asked to bring their children to the centre for a control visit every month, and every time their children experienced a febrile episode accompanied by symptoms suggesting an LRTI, such as cough, tachypnea, grunting, chest in-drawing, feeding difficulties, airway noise, irritability and poor sleep. The parents were also asked to complete a diary recording all of their children’s clinical problems and the administration of any drug including antipyretics. To overcome the problem of the possible under-reporting of wheezing episodes, all of the families were systematically telephoned weekly to verify the children’s status. All of the medical examinations (the planned monthly examinations and those following the development of a febrile episode) were carried out by trained investigators (SE and CT) using standardised questionnaires. At each visit, the details of any medical event occurring since the previous visit were recorded, and the children underwent a complete physical examination.

The controls were 119 age- and gender-matched healthy subjects with a negative history for LRTIs and wheezing, and no respiratory problems during their first two years of life, who were randomly selected on the basis of a computer-generated randomisation list from those attending the outpatient clinic for clinical control after minor surgical procedures during the study period. Although controls were clinically followed since the same age of the study patients, a 3 mL whole blood sample for genetic studies from them was obtained when they were aged 24 months in order to be sure that they did not suffer from wheezing episodes during the first years of life.

### Identification of viral infections

Viral RNA or DNA was extracted from the respiratory secretions by means of a Nuclisens EasyMAG automated extraction system (Biomeriéux, Craponne, France), and was then tested using the Luminex x TAG respiratory virus panel fast assay (Luminex Molecular Diagnostics Inc., Toronto, Canada) in order to detect influenza A virus (subtype H1 or H3), influenza B virus, RSV-A and -B, parainfluenzavirus-1, −2, −3 and −4, adenovirus, human metapneumovirus, coronaviruses 229E, NL63, OC43 and HKU1, enterovirus/RV and human bocavirus in accordance with the manufacturer’s instructions [13, 14]. The enterovirus/RV-positive samples were retested by means of a real-time polymerase chain reaction (RT-PCR) assay using the one-step iAg-Path-ID RT-PCR kit (Applied Biosystems, Foster City, CA), and the primers and probe sequences reported by Lu *et al*. [15] in order to identify the type of RV.

### Genetic studies

#### Candidate genes

Thirty-three candidate genes and 47 single nucleotide polymorphisms (SNPs) were selected for analysis, including genes involved in immune regulation, and the pathogenesis of inflammation and wheezing. The selected genes encode transcription (CHUK, IKIKB, JUN, NFKB1A, NFKB1B) and transduction factors (MAP3K1), the regulator of T cell activity (CTLA4), pattern recognition receptors (IL1RL1, TLR1, TLR3, TLR4, TLR5, TNFRSF1B), pro-inflammatory cytokines and related receptors (IL13, IL18, IL18RAP), anti-inflammatory cytokines (IL10, IL19, IL20), the cytokine regulating lymphoid cell survival (IL7), chemokines (CCL5, IL8, CXCL9), the regulator of IgE production (IL4R), collectins (MBL2), the protein responsible for the acute response to bacterial lipopolysaccharides (LBP), growth factors (NGF, VEGFA), the protein involved in the structural maintenance of chromosomes (RAD50), the spermatogenesis-associated serine-rich 2-like SPATS2L, the thromboxane A2 receptor (TBXA2R), and the adrenergic beta 2 surface receptor (ADRB2), and all are located on autosomes. In most cases, the SNPs were functional variants or tagging SNPs characterised by the International HapMap Project. The studied SNPs are involved in causing or determining the severity or outcome of wheezing in experimental animals or humans, or have previously been found to be associated with an increased risk of developing asthma or an abnormal immune response [16–47]. The investigated genes and SNPs are listed in Table 1.

**Table 1:** **Gene and single nucleotide polymorphisms (SNPs)**

Gene	dbSNP	HGVS description	Functional consequence	Position (bp)	Chr	Gene location
*ADRB2*	Rs1042713	NC_000005.9:g.148206440G > A	Mis-sense	148206440	5	Exon
*CCL5*	Rs2280788	NC_000017.10:g.34207405G > C	Upstream variant 2 KB	34207405	17	Intergenic
*CHUK*	Rs3808916	NC_000010.10:g.101947737C > T	Downstream variant	101947737	10	Intergenic
*CTLA4*	Rs3087243	NC_000002.11:g.204738919G > A	Downstream variant	204738919	2	Intergenic
*CXCL9*	Rs2276886	NC_000004.11:g.76928428C > T	Intron variant	76928428	4	Intron
*JUN*	Rs11688	NC_000001.10:g.59247993C > T	Transition substitution	59247993	1	Exon
*IKIKB*	Rs3747811	NC_000008.10:g.42129505A > T	Intron variant	42129505	8	Intron
	Rs10958713	NC_000008.10:g.42180716C > T	Intron variant	42180716	8	Intron
*IL1RL1*	Rs1921622	NC_000002.11:g.102966067G > A	Intron variant	102966067	2	Intron
*IL1R2*	Rs740044	NC_000002.11:g.102600169A > C	Transversion substitution	102600169	2	Exon
*IL4R*	Rs1805010	NC_000016.9:g.27356203A > G	Mis-sense	27356203	16	Exon
	Rs1805015	NC_000016.9:g.27374180 T > C	Mis-sense	27374180	16	Exon
	Rs1801275	NC_000016.9:g.27374400A > G	Mis-sense	27374400	16	Exon
*IL7*	Rs2583762	NC_000008.10:g.79697483A > T	Intron variant	79697483	8	Intron
*IL8*	Rs4073	NC_000004.11:g.74606024A > T	Upstream variant 2 KB	74606024	4	Intergenic
*IL10*	Rs1800872	NC_000001.10:g.206946407 T > G	Upstream variant 2 KB	206946407	1	Intergenic
	Rs1800896	NC_000001.10:g.206946897 T > C	Upstream variant 2 KB	206946897	1	Intergenic
*IL13*	Rs20541	NC_000005.9:g.131995964A > G	Mis-sense	131995964	5	Exon
	Rs1800925	NC_000005.9:g.131992809C > T	Upstream variant 2 KB	131992809	5	Intergenic
*IL18*	Rs187238	NC_000011.9:g.112034988C > G	Upstream variant 2 KB	112034988	11	Intragenic
*IL18RAP*	Rs917998	NC_000002.11:g.103068156C > T	Intron variant	103068156	2	Intron
*Il19*	Rs2243188	NC_000001.10:g.207014472A > C	Intron variant	207014472	1	Intron
	Rs2243191	NC_000001.10:g.207015957 T > C	Mis-sense	207015957	1	Exon
*IL20*	Rs2981573	NC_000001.10:g.207040577G > A	Intron variant	207040577	1	Intron
*LBP*	Rs12624843	NC_000020.10:g.36989096G > A	Intron variant	36989096	20	Intron
*MBL2*	Rs1800450	NC_000010.10:g.54531235C > T	Mis-sense	54531235	10	Exon
	Rs1800451	NC_000010.10:g.54531226C > T	Mis-sense	54531226	10	Exon
	Rs5030737	NC_000010.10:g.54531242G > A	Mis-sense	54531242	10	Exon
*MAP3K1*	Rs702680	NC_000005.9:g.56220382 T > C	Intron variant	56220382	5	Intron
	Rs889312	NC_000005.9:g.56031884C > A	Upstream variant	56031884	5	Intergenic
*NFKB1A*	Rs2233419	NC_000014.8:g.35871960G > A	Intron variant	35871960	14	Intron
	Rs3138052	NC_000014.8:g.35875021 T > C	Upstream variant 2 KB	35875021	14	Intergenic
*NFKB1B*	Rs3136641	NC_000019.9:g.39396649C > T	Intron variant	39396649	19	Intron
*NGF*	Rs11102930	NC_000001.10:g.115881055G > A	Upstream variant 2 KB	115881055	1	Intergenic
*RAD50*	Rs2706347	NC_000005.9:g.131905117G > T	Intron variant	131905117	5	Intron
*SPATS2L*	Rs295137	NC_000002.11:g.201150040C > T	Upstream variant	201150040	2	Intergenic
*TBXA2R*	Rs4523	NC_000019.9:g.3595794A > G	Synonymous codon	3595794	19	Exon
*TLR1*	Rs4543123	NC_000004.11:g.38792524A > G	Downstream variant	38792524	4	Intergenic
*TLR3*	Rs1519309	NC_000004.11:g.187015089C > T	Downstream variant	187015089	4	Intergenic
	Rs3775291	NC_000004.11:g.187004074C > T	Mis-sense	187004074	4	Exon
*TLR4*	Rs2737190	NC_000009.11:g.120464181G > A	Upstream variant	120464181	9	Intergenic
	Rs4986790	NC_000009.11:g.120475302A > G	Mis-sense	120475302	9	Exon
	Rs4986791	NC_000009.11:g.120475602C > T	Mis-sense	120475602	9	Exon
*TLR5*	Rs2241096	NC_000001.10:g.223310048G > A	Intron variant	223310048	5	Intron
*TNFRSF1B*	Rs1061622	NC_000001.10:g.12252955 T > G	Mis-sense	12252955	1	Exon
*VEGFA*	Rs833058	NC_000006.11:g.43731854C > T	Upstream variant	43731854	6	Intergenic
	Rs2146323	NC_000006.11:g.43745095C > A	Intron variant	43745095	6	Intron

*Chr* chromosome, *HGVS* Human Genome Variation Society - Genome Build 37.1 (http://www.ncbi.nlm.nih.gov); the position reflects the distance from short-arm telomere. *Bp* base pairs.

#### DNA extraction

DNA was extracted using the Masterpure DNA Purification kit (Epicentre, Madison, FL) in accordance with the manufacturer’s instructions, and a 50 μL final elution volume after purification. The extracted DNA was quantified using Picogreen reagent (Life Technologies, Monza, Italy) and an Infinite M200 PRO fluorimeter (Tecan Italia, Cernusco Sul Naviglio, Italy). Following the nucleic acid purification procedures, the samples were stored at −20°C until use.

#### Genotyping

Forty-seven SNPs in the 33 genes were genotyped using the Custom TaqMan® Array Microfluidic Cards genotyping system on an ABI 7900HT (Applied Biosystems, Foster City, CA). After PCR amplification, the alleles were detected by means of end-point analysis using SDS and TaqMan Genotyper software (Applied Biosystems, Foster City, CA). The data were entered into a Progeny database (Progeny Software, LLC, South Bend, IN) for the generation of datasets for analysis.

### Statistical analysis

A sample size of 119 subjects in each of the two groups achieved 80% power (with alpha = 0.05) to detect an unadjusted odds ratio (OR) of 2.1, assuming that in the control group the proportion of children with a given polymorphic allele for a given gene was 0.30. The test statistic used was the two-sided Mantel-Haenszel test.

The categorical data were compared between the groups using contingency table analysis with the χ^2^ or Fisher’s exact test, as appropriate. The continuous data were analysed using a two-sided Student’s t-test after checking that they were normally distributed (based on the Shapiro-Wilk statistic), or a two-sided Wilcoxon rank-sum test otherwise. Genotype frequencies were determined by means of direct counting. In order to investigate the Hardy-Weinberg equilibrium (HWE), the expected number of each genotype was compared with the observed number, and potential deviations were assessed using the chi-squared or likelihood ratio test, as appropriate. Age-adjusted OR and their 95% confidence intervals (CI) were calculated in order to measure the associations between selected SNPs and: 1) susceptibility to wheezing by comparing all of the children with wheezing with the controls; 2) susceptibility to recurrent wheezing by comparing the children who experienced more than one episode of wheezing during with those who did not. The data were controlled for multiple testing using the false discovery rate method (with the Benjamini-Hochberg procedure). All of the statistical analyses were made using SAS software, version 9.2 (Cary, NC, USA).

## Results

Nineteen children included in the group of patients were lost during the study period because parents refused the return every month to the study center and did not adequately collected information regarding respiratory infections of their children. Consequently, 119 patients were considered and compared to controls. Table 2 summarises the demographic and clinical characteristics of the study population. Gender and age distribution was similar between the children with wheezing and controls, and between the children with non-recurrent and recurrent wheezing. Most of the subjects were Caucasian, and all of them had been born at term. The duration of breastfeeding, use of pacifier and having at least one older sibling were similar between the groups, but exposure to cigarette smoke, high IgE levels and a family history of atopy were significantly more frequent among the children with wheezing than the controls (p = 0.002, p < 0.001 and p < 0.001), and a family history of atopy was significantly more frequent among the children with recurrent than those with non-recurrent wheezing (p = 0.001).

**Table 2:** **Demographic and clinical characteristics of the study population at the time of onset of first wheezing episode**

Characteristic	Control group (n = 119)	Children with wheezing (n = 119)	P-value	Children with non-recurrent wheezing (n = 45)	Children with recurrent wheezing (n = 74)	P-value
Females, n (%)	43 (36.1)	43 (36.1)	1.00	16 (35.6)	27 (36.5)	0.92
Median age (range), months	6 (0.3-12)	6 (0.3-12)	1.00	6 (0.3-12)	6 (0.3-12)	1.00
Caucasians, n (%)	113 (95.0)	113 (95.0)	1.00	42 (93.3)	71 (95.9)	0.67
Median gestational age (range), weeks	38.3 (37-41)	38.1 (37-41)	0.97	38.2 (37-41)	37.6 (37-40)	0.94
Subjects breastfed ≥3 months, n (%)	82 (68.9)	73 (61.3)	0.27	29 (64.4)	44 (59.5)	0.72
Subjects regularly using pacifier, n (%)	30 (25.2)	31 (26.1)	1.00	10 (22.2)	21 (28.4)	0.59
Subjects with at least one older sibling, n (%)	28 (23.5)	33 (27.7)	0.55	12 (26.7)	21 (28.4)	0.99
Subjects exposed to cigarette smoke, n (%)	55 (46.2)	79 (66.4)	0.002	26 (57.8)	53 (71.6)	0.17
Subjects with high IgE levels, n (%)	11 (9.2)	76 (63.9)	<0.001	24 (53.3)	52 (70.3)	0.09
Subjects with a family history of atopy, n (%)	24 (20.2)	85 (71.4)	<0.001	24 (53.3)	61 (82.4)	0.001

The selected SNP allele frequencies were similar in the children with any type of wheezing and in the controls, and similar in the children with non-recurrent or recurrent wheezing. All of the examined SNPs were present in the study population.

Table 3 shows the SNP genotype frequencies that were significantly different between the children with wheezing and that controls, and Table 4 those that were significantly different between the children with non-recurrent wheezing and those with recurrent wheezing. IL8-rs4073AT, VEGFA-rs833058CT, MBL2-rs1800450CT and IKBKB-rs3747811AT were associated with a significantly increased risk of developing wheezing (p = 0.02, p = 0.03, p = 0.05 and p = 0.0018), whereas CTLA4-rs3087243AG and NFKBIB-rs3136641TT were associated with a significantly reduced risk (p = 0.05 and p = 0.04). IL8-rs4073AT, VEGFA-rs2146323AA and NFKBIA-rs2233419AG were associated with a significantly increased risk of developing recurrent wheezing (p = 0.04, p = 0.04 and p = 0.03), whereas TLR3-rs3775291TC was associated with a significantly reduced risk (p = 0.03). No other differences in genotype frequencies were found.

**Table 3:** **Genotype frequencies of selected SNPs in controls and children with bronchospasm** ^**a**^

	Observed				Expected								
Gene and polymorphic alleles	Control group (n = 119)		Children with wheezing (n = 119)		Control group (n = 119)		Children with wheezing (n = 119)		HWE, ***χ*** ^2^ controls	HWE, ***χ*** ^2^ wheezing			
	N	%	N	%	N	%	N	%	p-value	p-value	OR ^b^	95% CI	p-value ^c^
**IL8-rs4073**													
**A**	28	23.7	21	17.8	22.5	19.0	20.8	17.6			1.00	(Reference)	-
**A/T**	47	39.8	57	48.3	58.0	49.2	57.5	48.7			2.50	(1.15-5.44)	0.02
**T**	43	36.4	40	33.9	37.5	31.8	39.8	33.7	0.04	0.93	1.57	(0.71-3.45)	0.27
**VEGFA-rs833058**													
**C**	32	27.1	22	18.5	32.6	27.6	28.3	23.8			1.00	(Reference)	-
**C/T**	60	50.9	72	60.5	58.8	49.9	59.5	50.0			2.22	(1.09-4.55)	0.03
**T**	26	22.0	25	21.0	26.6	22.5	31.3	26.3	0.83	0.02	1.96	(0.83-4.65)	0.13
**MBL2-rs1800450**													
**C**	87	73.7	76	63.9	87.3	74.0	77.4	65.1			1.00	(Reference)	-
**C/T**	29	24.6	40	33.6	28.4	24.1	37.1	31.2			1.90	(1.01-3.58)	0.05
**T**	2	1.7	3	2.5	2.3	2.0	4.4	3.7	0.81	0.40	1.03	(0.15-7.23)	0.97
**IKBKB-rs3747811**													
**A**	36	31.3	20	17.1	28.3	24.6	24.5	20.9			1.00	(Reference)	-
**A/T**	42	36.5	67	57.3	57.5	50.0	58.1	49.6			3.26	(1.55-6.85)	0.0018
**T**	37	32.2	30	25.6	29.3	25.4	34.5	29.5	0.004	0.10	2.01	(0.89-4.53)	0.09
**CTLA4-rs3087243**													
**A**	25	21.2	35	29.7	32.1	27.2	35.8	30.3			1.00	(Reference)	-
**A/G**	73	61.9	60	50.9	58.9	49.9	58.4	49.5			0.50	(0.25-0.99)	0.05
**G**	20	17.0	23	19.5	27.1	22.9	23.8	20.2	0.009	0.76	0.65	(0.27-1.56)	0.34
**NFKBIB-rs3136641**													
**C**	64	54.2	72	61.0	59.8	50.7	71.0	60.1			1.00	(Reference)	-
**C/T**	40	33.9	39	33.1	48.4	41.0	41.1	34.8			0.90	(0.49-1.66)	0.73
**T**	14	11.9	7	5.9	9.8	8.3	6.0	5.0	0.06	0.58	0.33	(0.11-0.94)	0.04

^a^The sums may not add up to the total because of a few missing values; ^b^Odds ratios adjusted for age (< / ≥ 2 years). ^c^p-values from univariate analyses, not adjusted for multiple testing. None of the p-values was significant after correction for multiple testing.

*HWE* Hardy-Weinberg equilibrium, *NE* Not estimable.

**Table 4:** **Genotype frequencies of selected SNPS with significant differences in children with recurrent or non-recurrent wheezing** ^**a**^

	Observed				Expected								
Gene and polymorphic alleles	Non-recurrent (n = 74)		Recurrent wheezing (n = 45)		Non-recurrent (n = 74)		Recurrent wheezing (n = 45)		HWE, ***χ*** ^2^ non-recurrent	HWE, ***χ*** ^2^ recurrent			
	N	%	N	%	N	%	N	%	p-value	p-value	OR ^b^	95% CI	p-value ^c^
**IL8-rs4073**													
**A**	17	23.3	4	8.9	12.3	16.9	8.5	18.8			1.00	(Reference)	-
**A/T**	26	35.6	31	68.9	35.3	48.4	22.1	49.1			3.60	(1.03-12.57)	0.04
**T**	30	41.1	10	22.2	25.3	34.7	14.5	32.1	0.02	0.007	1.18	(0.31-4.51)	0.81
**VEGFA-rs2146323**													
**A**	4	5.4	8	17.8	6.8	9.2	6.8	15.1			4.32	(1.06-17.64)	0.04
**A/C**	37	50.0	19	42.2	31.3	42.3	21.4	47.5			1.08	(0.46-2.54)	0.85
**C**	33	44.6	18	40.0	35.8	48.4	16.8	37.3	0.12	0.45	1.00	(Reference)	-
**TLR3-rs3775291**													
**T**	10	13.7	4	8.9	8.9	12.2	2.5	5.4			0.75	(0.20-2.75)	0.66
**T/C**	31	42.5	13	28.9	33.2	45.5	16.1	35.8			0.36	(0.14-0.90)	0.03
**C**	32	43.8	28	62.2	30.9	42.3	26.5	58.8	0.57	0.20	1.00	(Reference)	-
**NFKBIA-rs2233419**													
**A**	5	6.8	2	4.4	1.9	2.6	2.2	4.9			0.82	(0.13-5.05)	0.83
**A/G**	14	18.9	16	35.6	20.1	27.2	15.6	34.6			2.79	(1.12-6.99)	0.03
**G**	55	74.3	27	60.0	51.9	70.2	27.2	60.5	0.009	0.85	1.00	(Reference)	-

^a^The sums may not add up to the total because of a few missing values; ^b^Odds ratios adjusted for age (< / ≥ 2 years). ^c^p-values from univariate analyses, not adjusted for multiple testing. None of the p-values was significant after correction for multiple testing. *HWE* Hardy-Weinberg equilibrium.

Table 5 shows the distribution of viral infections in the children with non-recurrent or recurrent wheezing. RSV was the most frequently detected pathogen in the majority of the study population, followed by RV. The frequency of RSV was significantly higher in patients with non-recurrent wheezing than in those with recurrent episodes (p = 0.02). Interestingly, the study of gene-environment interactions showed that RV was significantly associated with recurrent wheezing in the presence of IL4Ra-rs1801275GG and G (OR 6.03, 95% CI: 1.21-30.10, p = 0.03) and MAP3K1-rs702689AA (OR 4.09, 95% CI: 1.14-14.61, p = 0.03). No significant difference was observed in the distribution of genetic polymorphisms among the children with RSV or other viral infections and non-recurrent or recurrent wheezing.

**Table 5:** **Frequency distribution of viral infections in children with recurrent or non-recurrent wheezing**

Viral infection	Non-recurrent wheezing (n = 74)		Recurrent wheezing (n = 45)		
	N	%	N	%	p-value
**RSV**					
**No**	21	28.4	22	48.9	
**Yes**	53	71.6	23	51.1	**0.02**
**RV (any type)**					
**No**	59	79.7	33	73.3	
**Yes**	15	20.3	12	26.7	0.42
**Enterovirus**					
**No**	70	94.6	37	82.2	
**Yes**	4	5.4	8	17.8	0.055
**Coronavirus (any type)**					
**No**	71	96.0	43	95.6	
**Yes**	3	4.0	2	4.4	1.00
**Metapneumovirus**					
**No**	68	91.9	43	95.6	
**Yes**	6	8.1	2	4.4	0.71
**Bocavirus**					
**No**	72	97.3	43	95.6	
**Yes**	2	2.7	2	4.4	0.63
**Influenza (any type)**					
**No**	72	97.3	44	97.8	
**Yes**	2	2.7	1	2.2	1.00
**Parainfluenza (any type)**					
**No**	72	97.3	45	100.0	
**Yes**	2	2.7	0	0.0	0.53
**Any viral infection**					
**No**	7	9.5	8	17.8	
**Yes**	67	90.5	37	82.2	0.18

p-values from chi-squared or Fisher’s exact test, as appropriate. *RSV* respiratory syncytial virus, *RV* rhinovirus. The frequency of RSV was significantly higher in patients with non-recurrent wheezing than in those with recurrent episodes (p = 0.02).

## Discussion

This study shows that genetic polymorphisms seem to play a substantial role in conditioning the risk of wheezing in young children with viral LRTI. Susceptibility to the development of wheezing seems to be greater in children with SNPs of the IL8 (rs4073AT), VEGFA (rs833058CT), MBL2 (rs1800450CT) and IKBKB genes (rs3747811AT). The association with IL8, VEGFA and MBL2 genes is not surprising because the factors encoded by them play an important role in immunity and have been previously associated with bronchial obstruction [8–11].

IL8 is one of the most important chemotactic factors for leukocyte recruitment and activation in the lung, and has to be controlled to prevent excessive tissue injury and possibly damaging systemic effects [1]. The SNP of IL8 evaluated in this study has previously been found to be significantly associated with severe RSV bronchiolitis and recurrent wheezing episodes [22], and it has also been found that a high concentration of IL-8 in cord blood is significantly associated with persistent wheezing at the age of one year [23]. Its importance in conditioning susceptibility to wheezing seems to confirmed by the finding that it was the only genetic polymorphism associated with the risk of wheezing and susceptibility to recurrent episodes.

VEGFA is a major regulator of angiogenesis and enhancer of vascular permeability [24], and seems to induce airway inflammation and airway remodelling in patients with asthma [25]. High VEGFA levels have been found in subjects with chronic pulmonary diseases [26] and certain kinds of pneumonia [27], and have been found to be significantly higher in the serum of children with *Mycoplasma pneumoniae* infection and wheezing than in healthy controls [28].

MBL2 is a protein belonging to the collectin family that is important in the innate immune system [29]. It recognises mannose and N-acetylglucosamine on many micro-organisms, and is capable of activating the classical complement pathway. Deficiencies in this gene have been associated with susceptibility to autoimmune and infectious diseases [29], and a well-defined but weak association has been found between some SNPs of MBL2 and asthma and atopy in children [29].

There is no previous report of an association between the IKBKB gene and increased susceptibility to wheezing. This gene encodes IkB kinase 2 (IKK2, also known as IKKβ), a component of the IKK–nuclear factor κB (NF-κB) pathway [30]. A number of primary immunodeficiencies leasing to an increased risk of infection and autoimmunity are caused by an impaired IKK–IκB axis [30] and, as IKKs are required for the IL17-dependent signalling associated with neutrophilia and pulmonary inflammation [31], and IL17 levels have been found to be higher in patients with asthma [32], it is possible that SNPs of IKBKB could lead to an increased risk of wheezing.

A reduced risk of developing wheezing was found in the children with CTLA4 and NFKBIB SNPs, and TLR3 SNP was associated with a reduced risk of developing recurrent wheezing. The data concerning NFKBIB, a factor involved in innate immunity, conflict with those collected by Daley *et al*. [10] and Sharma *et al*. [11], who studied a large number of SNPs and found that children with a genetic abnormality in NFKBIB and viral respiratory infection in early life were at increased risk of developing asthma. The data concerning CTLA4 are in line with the findings of some authors [34], but not others [35]. This factor plays an essential role in the function of the regulatory T cells that control Th1 and Th2 immune responses [33], and Borrego *et al*. have reported that, in comparison with healthy children, CTLA4 expression is diminished in wheezy subjects and those at high risk of asthma, thus suggesting that impaired CTLA4 expression may play a role in reducing bronchial obstruction [34]. However, Ermers *et al*. found that CTLA43087243AG was significantly more frequent in children with wheezing than in healthy controls [35].

TLR3 recognises dsRNA derived from respiratory viruses and activates nuclear factor-κB, which increases interferon-γ signalling in other cells and transforms airway inflammation into asthma [36]. Th2 cytokines participate in this process by modulating the response of airway epithelium and dendritic cells to dsRNA11 and, in the context of RV infection, the binding of viral dsRNA to TLR3 initiates pro-inflammatory signalling pathways that lead to airway inflammation and hyper-responsiveness [37]. Contrary to our own findings, Nuolivirta *et al*. found that TLR3-rs3752291TC was associated with an increased risk of bronchiolitis and that TLR3-rs3752291CC was significantly more frequent in patients with recurrent wheezing [38]. Moreover, Daley *et al*. documented a positive association between TLR3 SNPs and atopic asthma [10]. These discrepancies may be due to differences in the ethnicity and general characteristics of the study populations, such as differences in wheezing phenotypes, because it has been found that recurrent wheezing during the first year after viral LRTI and recurrent wheezing at the age of six years are distinct entities with their own specific immunological and genetic characteristics [39]. It also possible that the results were influenced by gene-gene interactions and/or differences in HWE.

Our findings regarding the associations between SNPs and gene-environment interactions are also partially different from those of previous studies. As expected, RSV was the most frequently detected pathogen in our study, and its frequency was significantly higher in children with non-recurrent wheezing than in those with recurrent episodes. This may be explained by the fact that the vast majority of our patients were affected by RSV infection, but there was no difference in the distribution of genetic polymorphisms between the children with recurrent or non-recurrent episodes due to RSV infection.

On the other hand, RV infection was significantly associated with recurrent wheezing in the presence of SNPs in the IL4Ra and MAP3K1 genes. The IL4Ra gene encodes the alpha chain of the interleukin-4 receptor, a type I transmembrane protein that can bind IL4 and IL13 and thus regulate IgE antibody production in B cells [40], and the encoded protein can also bind IL4 in T cells to promote differentiation of Th2 cells. Allelic variations in this gene have been associated with atopy and allergic clinical manifestations [40]. Furthermore, RV infections are a major cause of asthma exacerbations [41, 42], and RVs have also been repeatedly detected in patients with stable asthma [43]. A number of studies have shown abnormalities in innate immune responses to viruses in patients with asthma, particularly deficiencies in the production of IFN I and III [44–46]. The finding that a SNP in the IL4Ra gene is associated with recurrent wheezing episodes during RV LRTI further contributes to explaining the complications that could follow RV infection.

Similar conclusions can be drawn in relation to the association between the SNP in the MAP3K1 gene and wheezing recurrences in children with RV infection. Our data extend the findings of Szczepankiewicz *et al*., who observed an association between asthma and MAP3K1 gene polymorphisms rs702689 and rs889312, and suggested that genetic variants of the MAP3K1 gene involved in regulating neurogenic inflammation may contribute to asthma possibly via enhanced NGF expression and the activation of the MAPK signalling pathway [47].

Our study has some limitations. First of all, the number of enrolled subjects was relatively small and further studies of larger pediatric populations would increase the probability that the statistical associations found by us represent true biological differences and reduce the risk of false positive results. Secondly, although considerable, the number of studied genes and SNPs was probably less than the number of genes and genetic variations actually involved in regulating the mechanisms that play a role in conditioning susceptibility to infection and the development of wheezing. Finally, we did not evaluate the effect of common environmental exposure on the associations between the genes of the immune pathway and wheezing but, as it has been suggested that environmental exposure may modify the effect of such genetic variants on disease susceptibility, an analysis of gene-environmental interactions is necessary before drawing any definite conclusions.

## Conclusions

Our findings show a clear relationship between the risk of wheezing and polymorphisms of some of the genes involved in the immune response. Despite not conclusive, they indicate that genetics play an important role in conditioning wheezing development. However, given the conflicting literature, further large studies are needed in order to establish which are the real associations between wheezing development and genetic variations.

## Abbreviations

CI: 95% confidence intervals
HWE: Hardy-Weinberg equilibrium
LRTI: Lower respiratory tract infection
ORs: Odds ratios
RSV: Respiratory syncytial virus
RV: Rhinovirus
PCR: Polymerase chain reaction
SNPs: Single nucleotide polymorphisms.
